# Synthesis and Mass Spectrometry Structural Assessment of Polyesteramides Based on ε-Caprolactone and L-Phenylalanine

**DOI:** 10.3390/polym16212955

**Published:** 2024-10-22

**Authors:** Slim Salhi, Houcine Ammar, Joanna Rydz, Cristian Peptu

**Affiliations:** 1 Laboratoire de Chimie Appliquée, Faculté des Sciences de Sfax, Université de Sfax, Sfax 3000, Tunisia; houcine.ammar@fss.usf.tn; 2 Centre of Polymer and Carbon Materials, Polish Academy of Sciences, M. Curie-Skłodowska 34, 41-819 Zabrze, Poland; jrydz@cmpw-pan.pl; 3 Polish-Romanian Laboratory ADVAPOL, M. Curie-Skłodowska 34, 41-819 Zabrze, Poland; 4 Petru Poni Institute of Macromolecular Chemistry, Grigore Ghica Voda Alley, 41A, 700487 Iasi, Romania

**Keywords:** polyesteramide, ε-caprolactone, L-phenylalanine, melt polymerization, MALDI MS, MALDI MS/MS

## Abstract

L-Phenylalanine-ε-caprolactone-based polyesteramides (PCPs) were synthesized via melt polycondensation across a diverse range of molar compositions. The copolymer structure was extensively characterized using nuclear magnetic resonance (NMR) and matrix-assisted laser desorption/ionization mass spectrometry (MALDI MS). NMR analysis confirmed the intercalation of the L-Phenylalanine comonomer units within the polyester backbone. MALDI MS characterization further demonstrated the formation of linear PCP chains with carboxyl end groups. A detailed structural analysis through MALDI MS/MS fragmentation indicated that ester bond scission was the predominant fragmentation mechanism, depicting the polyesteramide sequence in the copolymers. The resulting copolymers were primarily amorphous, except for those with molar compositions of 90/10 and 80/20, which exhibited semi-crystalline structures. Additionally, these PCPs showed an increase in glass transition temperatures with higher amino acid contents and demonstrated good thermal stabilities, as evidenced by a 10% mass loss at elevated temperatures.

## 1. Introduction

During the past few decades, biodegradable and biocompatible polymeric materials for biomedical or environmental applications have attracted considerable attention from researchers around the world [1,2].

Significant work has featured the synthesis of aliphatic polyesters, such as polycaprolactone (PCL) [3,4,5], polylactide (PLA) [6,7,8,9], and poly(lactic-co-glycolic acid) (PLGA) [10,11,12], or their copolymers [10,13,14,15]. The polyesters exhibit great flexibility, high elasticity, and controllable biodegradability. Currently, polymer degradability is a major parameter for polymeric products addressing an extensive range of applications. However, they possess an insufficiency of hygroscopicity and hydrophilicity and do not fulfill the requirements of some other applications [16,17].

On the other hand, polyamides (PAs) have been extensively used in the field of chemical engineering, specifically in the textiles and light industry [18,19]. PAs containing amide bonds between each monomer usually show high mechanical endurability and thermal stability. However, they are slowly biodegrading due to intermolecular hydrogen bonding interactions.

Therefore, combining these two classes of polymers into a single polymer entity seems attractive. The resulting polymer polyesteramides (PEAs) can virtually combine the advantages of the two previously mentioned polymers, thereby associating the stiffness and high mechanical and thermal properties of polyamides with the biodegradability and biocompatibility of polyesters [20,21]. In 1932, Carothers synthesized the first PEA from diacids, diols, and diamines [22].

Among natural models and artificial processing feasibility, amino acids have been used in the synthesis of a new pseudoprotein PEA, shown to be a breakthrough in creating a PEA which is more relevant to the synthetic and degradable polymers used in the biomedical fields [23,24]. Amino acid-based polyesteramides (AA-PEAs) combine the valuable qualities of traditional PEAs with the benefits of the existence of α-AAs which can improve their use in some applications such as drug delivery, the development of cell–material interactions, and enzymatic degradability [25,26,27].

Several architectures of AA-PEAs have been synthesized resulting in block [28], alternating [29], or random [30] copolymers. Block PEA structures may employ the ring-opening polymerization (ROP) of cyclic esters such as ε-caprolactone (CL) starting from an oligopeptide sequence or AA-based cyclic compounds, such as the α-amino acid N-carboxy anhydrides which may be polymerized starting from oligoester sequences [31,32,33]. On the other hand, the synthesis of alternating structures may use various strategies, e.g., coupling reactions of diacid compounds with diamine-terminated diesters [34,35,36,37]. The alternating structure may also be synthesized through the ring-opening polymerization of morpholine-2,5-diones [38,39]. The obtained polymers, known as polydepsipeptides, are composed of intercalated α-amino and α-hydroxy acids [25,40,41]. However, synthesizing this kind of cyclic compound from larger monomers is not a trivial task, thereby limiting this method to the preparation of PEAs from α-amino acids and α-hydroxy acids. Furthermore, this simple procedure to prepare random PEAs does not demand the use of solvents or expensive and sensitive monomers like N-carboxy anhydrides. Later research proposed the simultaneous synthesis of morpholine-2,5-diones from AA mixtures that may be found in protein-rich waste, thus leading to the synthesis of PEA copolymers [42].

Recently, the synthesis of PEAs has been reported via the direct reaction of amino acids with cyclic esters or by the direct bulk polycondensation of amino acids and α-hydroxy acids [43,44,45,46,47]. Thus, some of us proposed a synthesis method for polyesteramides with random comonomer sequences using β-alanine [46,48] or L-alanine [47]. L-phenylalanine (Phe) was used to prepare bioabsorbable polyesteramides due to various reasons. It is classified as an essential α-amino acid among others that can be, after the polymer’s degradation, assimilated by some living organisms. Also, L-phe has a lateral benzyl group which is recognizable by some enzymes [49]. Moreover, poly(ester-adipamide)s which are based on ethylenic diesters of bis(α-amino acids) and L-phe underwent significant in vitro degradation when compared to other hydrophobic α-amino acids. Therefore, polymers which include L-phe in the main chain are appropriate for biodegradation owing to the relation between their susceptibility towards biodegradation and their chemical structure [50]. Subsequently, PEAs based on L-aminoacids that possess symmetrical side substituents such as Val, Leu, and Phe had higher Tg’s than those from nonsymmetrical side substituents. Consequently, L-Phe represents an interesting amino acid used to synthesize polyesteramides [51,52,53].

Herein, the synthesis of PEAs starting from ε-CL and Phe by melt polycondensation with a range of molar compositions is reported. The structural characterization of the obtained products is performed through a deep study comprising NMR, MALDI MS, and MS/MS characterization to analyze the ester/amide bond sequences. Further, TGA and DSC analyses are employed to establish the composition influence on the thermal properties of the prepared PEA.

## 2. Materials and Methods

### 2.1. Materials

L-Phenylalanine (Phe, >98%), ε-caprolactone (CL, >97%), Tetrabutoxytitanium (Ti(OBu)_4_, >97%), and trifluoroacetic anhydride (TFA, >99%) were purchased from Sigma-Aldrich (Saint Louis, MO, USA) and used as received.

### 2.2. Synthesis of Poly(ε-caprolactone-phenylalanine) (PCP-x/y)

The synthesis of different PCP-x/y structures, where x/y represents the CL/Phe molar ratio in the reaction feed, was carried out via direct melt polycondensation. For instance, PCP-70/30 was prepared as follows: a mixture of CL (10 g, 0.087 mol) and Phe (6.20 g, 0.037 mol) was heated at 200 °C under nitrogen for 1 h in a 200 mL reactor fitted with a mechanical stirrer, nitrogen inlet, and a vacuum line. A 0.2 wt% of tetrabutoxytitanium was accurately added and the mixture was allowed to react for another hour, at 200 °C. The reactor was then heated to 240 °C for 4 h under vacuum (0.01 mmHg). All resulting polymers were analyzed without further purification.

### 2.3. Synthesis of Poly(ε-caprolactone) (PCL)

PCL was synthesized by the ROP of CL, employing similar conditions to those used for PEA synthesis. A 20.0 g CL amount was heated at 200 °C together with a 0.2 wt% of Ti(OBu)_4_. Initially, the monomer was heated for 1 h under a nitrogen atmosphere, followed by the application of a vacuum for an additional 2 h.

### 2.4. Characterization Methods

^1^H and ^13^C nuclear magnetic resonance (NMR) spectroscopy analyses of samples dissolved in CDCl_3_/TFA solutions (2/1 *v*/*v*) were recorded on a Bruker Avance 400 spectrometer (Bruker, Rheinstetten, Germany) at 400 MHz (^1^H NMR) or 100 MHz (^13^C NMR) using a 5 mm inverse probe. Chemical shifts were referenced to the CDCl3 peak at 7.26 ppm (^1^H) and the central peak at 77.16 ppm (^13^C).

MALDI MS characterization of the polymer samples employed a RapifleX MALDI TOF/TOF MS (Bruker, Bremen, Germany) controlled with FlexControl 4.0 and FlexAnalysis 4.0 software (Bruker, Bremen, Germany). Tetrahydrofuran was used to dissolve the samples at a concentration of 20 mg/mL. The trans-2-[3-(4-tert-Butylphenyl)-2-methyl-2-propenylidene] malononitrile matrix solution (DCTB) was also prepared in THF solvent at a 20 mg/mL concentration. To promote a better sample ionization in MALDI conditions the samples were spiked with NaI solutions at 5 mg/mL concentrations. The above-mentioned solutions were mixed (2 μL of sample solution, 20 μL DCTB matrix, and 2 μL of NaI) and 1 μL was left to dry in room conditions on the MALDI plate. The mass spectra were obtained working in positive mode and the laser power was set just above the minimum value necessary to obtain a clear signal. The poly(ethylene glycol)/DCTB was used for MS calibration. The MS/MS fragmentation profiles were obtained in LIFT mode.

Attenuated total reflection Fourier transform infrared (ATR FTIR) analysis was performed with a PerkinElmer spectrum 100 spectrometer (Shelton, CT, USA) in the 4000–700 cm^−1^ range.

DSC thermograms were obtained with a TA Instruments DSC Q2000 system (New Castle, DE, USA), under nitrogen with cooling and heating rates as follows: 10 °C/min in the temperature range of −80 to 200 °C. The samples were subjected to two successive temperature ramps. TRIOS software (v5.1.1.46572) was employed for analysis.

Thermogravimetric analyses (TGAs) were carried out using a TA Instruments TGA Q500 (New Castle, DE, USA). Samples weighing between 3 and 8 mg were placed in a platinum crucible and heated from 20 °C to 800 °C at a rate of 10 °C per minute under a nitrogen atmosphere.

SEC measurements (*Mn* and *Mw* and *Đ*) were performed on the sample solutions in THF (10 mg mL^−1^) using linear polystyrene calibration standards. The employed Shimadzu LC-20AD system (Kyoto, Japan) consisted of two Varian PL gel 5 μm MIXED-C columns (all elements kept at a 30 °C constant temperature, 100 μL injection volume) with a refractive index detector (Shimadzu RID-10A). The THF solvent flow rate was set at 1.0 mL min^−1^.

## 3. Results

### 3.1. Synthesis of Poly(ε-caprolactone-phenylalanine) (PCP-x/y)

**Scheme 1:**
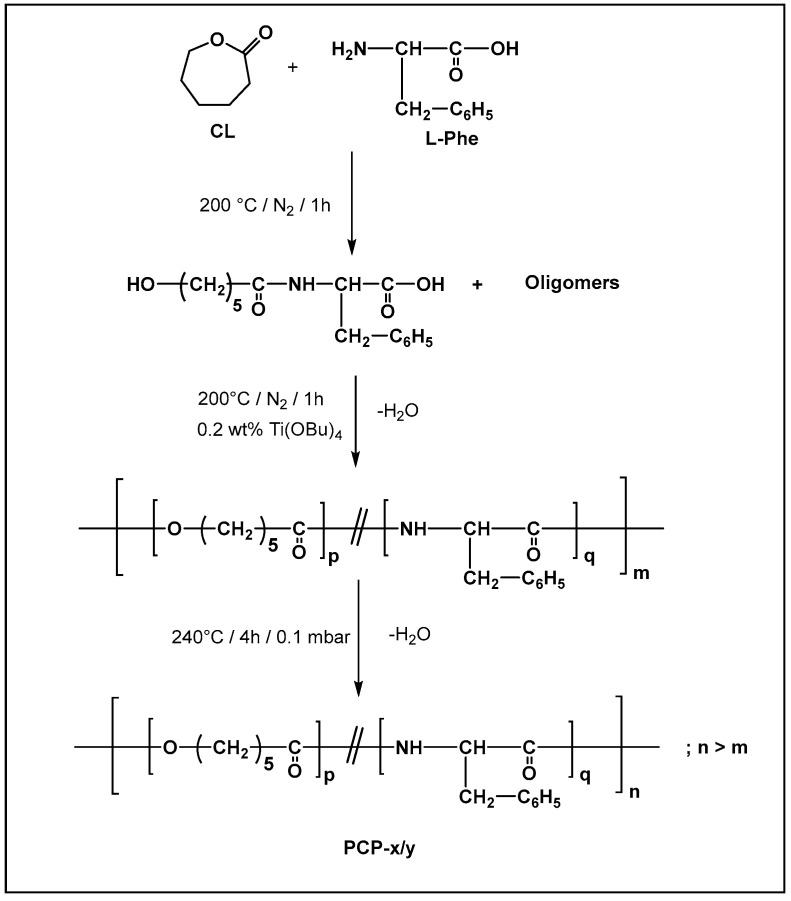
Synthesis of the PCP-x/y copolymer.

Poly[(ε-caprolactone)-(L-phenylalanine)] copolymers (PCPs) were synthesized from ε-CL and Phe through melt polycondensation, as depicted in Scheme 1. To obtain polyesteramides based on CL and Phe, mixtures of these monomers in various molar ratios (Table 1) were reacted in the presence of tetrabutoxytitanium to facilitate dimer formation. This process resulted in oligomers with either hydroxy and carboxy end groups or amino and carboxy end groups, depending on which functional group of the amino acid (amine or carboxyl) first reacts with ε-CL. The resulting oligomer mixture was then subjected to a vacuum to remove polycondensation water, thereby producing the corresponding polyesteramides.

**Scheme 2:**
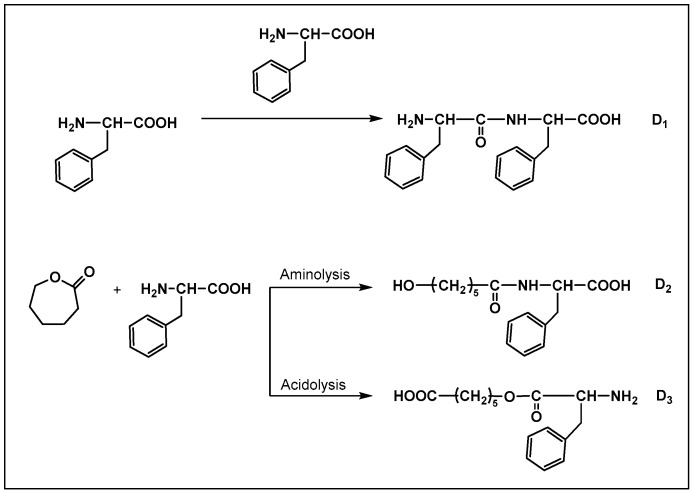
Possible reactions between comonomers affecting the chain sequence.

The structure of the prepared oligomers was influenced by the reactions that occurred during the initial heating step. Thus, three types of compounds may be hypothesized as follows: D1—formed through Phe dimerization, D2—CL aminolysis, and D3—CL acidolysis, respectively (Scheme 2).

Further, the reaction system led to the formation of PEA chains that largely depended on the initially formed CL/Phe sequences. The PCL homopolymer was found to have the highest *Mw,* according to the SEC analysis shown in Table 1. Also, the increasing Phe amount in the reaction feed environment seemed to negatively influence the polymer chain growth. Increasing the Phe fraction in the initial mixture led to PCPs with decreasing molar masses. Other reaction systems such as (ε-caprolactone-co-β-alanine) [45], (ε-caprolactone-co-glycine) [46], and (ε-caprolactone-co-L-alanine) [47] revealed a similar trend. Therefore, we may hypothesize that such behavior may be triggered by a catalyst inactivation via interactions between the amine or amide groups of amino acid units and the titanium esterification catalyst.

**Figure 1:**
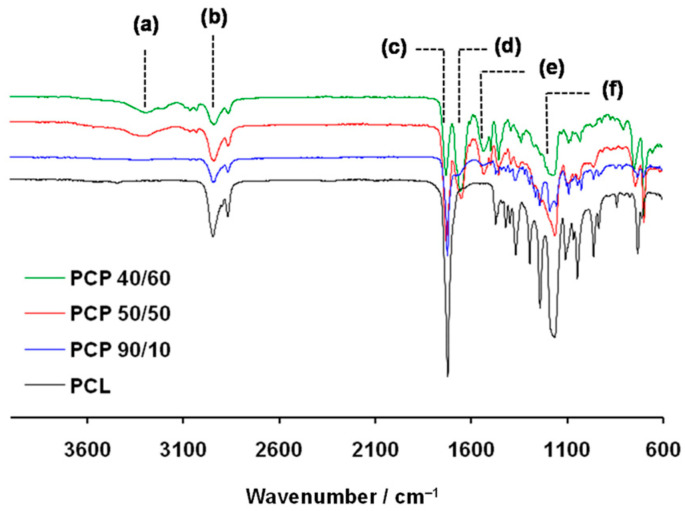
FTIR spectra of PCL and the obtained PCP-x/y polyesteramides.

The initial FTIR structural analysis confirmed the formation of the PCP-x/y copolymers as suggested by the observation of the characteristic bands as follows: amide NH (a), amide I (d), and amide II (e) at 3315, 1666, and 1535 cm^−1^, respectively (Figure 1). Additionally, absorption bands for the aliphatic CH (b) at 2942 cm^−1^, ester carbonyl (c) at 1734 cm^−1^, and C–O simple bond at 1167 cm^−1^ (band f) were also identified. The analyzed spectra confirmed the expected trend in comonomer content, with the increasing intensity of the amide I band (d) being directly correlated with the higher Phe ratio in the reaction feed.

### 3.2. Structural Characterization

**Figure 2:**
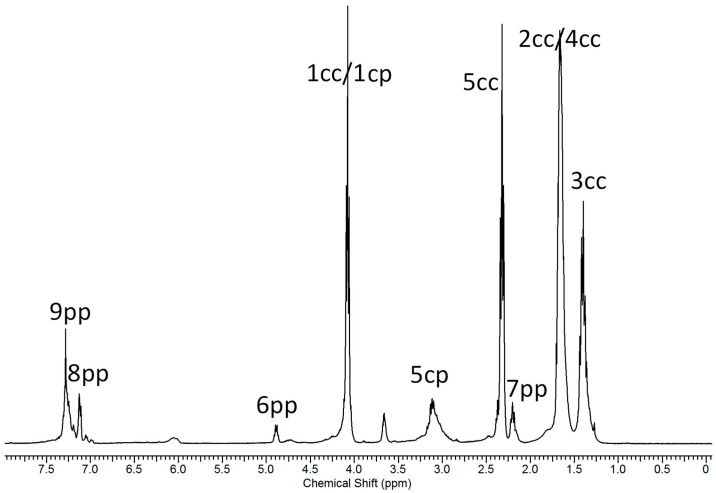
^1^H NMR spectrum of PCP-70/30 (400 MHz, CDCl_3_; ref δ(CHCl_3_) = 7.26 ppm).

The ^1^H NMR and ^13^C NMR spectra of PCP-x/y were further employed for structural evaluations. The ^1^H NMR spectrum of PCP-70/30 is given in Figure 2. The comonomer sequences were described as triads composed of combinations of CL (annotated with C) and Phe (annotated with P) comonomers. For ease of a structural description, Table 2 represents the observed comonomer neighboring triads, based on the NMR characterization and the atom numbering labels. The signals observed in the ^1^H NMR spectrum at 4.09 ppm and 2.32 ppm, respectively, were associated with the CL methylene protons labeled 1cc (ε) and 5cc (α), which may be found in the CCC, CCP, and PCC triads. The 1cp signal observed at 4.09 ppm (overlapping with 1cc) corresponded to the ε methylene of the CL units in PC dyads. The neighboring relationship between the Phe and CL comonomers was suggested by the presence of two broad signals at 3.07 ppm and 4.90 ppm, assigned to the α methylenes of CL (5cp) and methylenes of Phe units (6cp) in the CCP and PCP triads. The proton resonances of the three β, γ, and δ methylenes of CL units (signals annotated 2c, 3c, and 4c) were observed at 1.64 ppm (2c and 4c) and 1.39 ppm (3c), respectively. The mixed sequences did not affect the characteristic shift of these signals.

The PP dyads gave three signals. The first signal, found at 2.19 ppm, was assigned to the methylene protons (7ppp) from the PPP and PPC triads. Another two signals corresponded to the PPP triads: at 4.90 ppm (6ppp) and the protons of the phenyl ring at 7.12 and 7.28 ppm, respectively. However, the signals assigned to the Phe protons were broadened probably because of the various sequences such as CPP, PPC, and CPC, respectively. Thus, although the spectrum were annotated only with the signals belonging to pp sequences, the Phe correlated signals also reflected the CL intercalated sequences such as cpc, cpp, or ppc. ^1^H NMR peak broadening and overlapping prevented further *M_n_* calculations.

**Figure 3:**
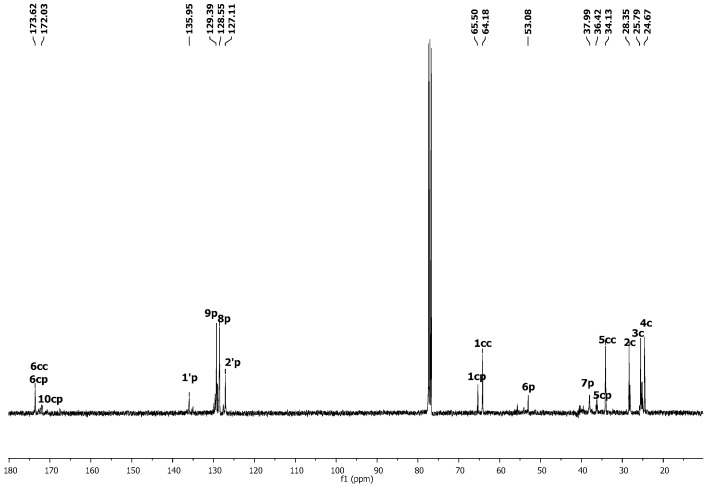
^13^C NMR spectrum of PCP-70/30 (400 MHz, CDCl_3_; ref δ (CDCl_3_) = 77.16 ppm).

The ^13^C NMR spectrum of PCP 70/30 (Figure 3) was also examined to establish the chemical shifts associated with the copolymer sequences. First, three signals corresponding to the carbonyl functions were noticed in the 160–180 ppm interval as follows: carbon signals associated with the carbonyl (ester) from CC dyads (6cc) at 173.62 ppm, amide carbonyls of CP dyads and carbonyl (ester) of mixed CPC and PPC triads (6cp and 10cp, respectively) for the broad signal around 172.03 ppm. In the 30–70 ppm range, the signals of CC dyads (1cc and 5cc at 64.18 and 34.13 ppm) and PC and CP dyads (1cp and 5cp at 65.50 and 36.42 ppm) were apparent from the ^13^C NMR analysis. As remarked for the proton NMR, the resonances of the β, γ, and δ methylene carbon atoms of CL units were not affected by the Phe neighboring, and thus the 2c, 3c, and 4c signals appeared at 28.38, 25.79, and 24.67 ppm, respectively. Similarly, the resonance of the Phe methylene carbon was not affected by the nature of neighboring units and appeared at 53.08 ppm. The carbon resonances belonging to the benzyl group 2p’, 8p, 9p, and 1’p appeared at 127.11, 128.55, 129.39, and 135.95 ppm, respectively. ^13^C NMR allowed differentiation of 1cc and 1cp signals which appeared to overlap in the ^1^H NMR.

### 3.3. MALDI MS Characterization

Matrix-assisted laser desorption/ionization mass spectrometry (MALDI MS) was further used to analyze the PCP copolymers. This technique provided detailed information on the chain composition of the synthesized copolymers. By analyzing the mass spectra, the presence of different oligomeric species could be identified, along with insights into the copolymer’s chain length and the nature of its terminal groups.

**Figure 4:**
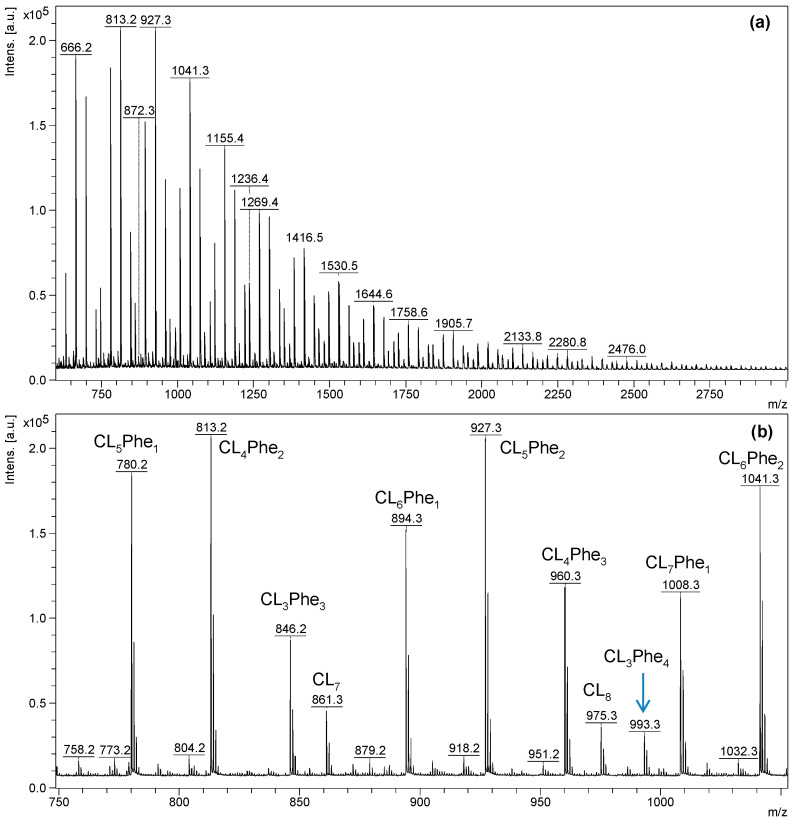
MALDI MS spectrum of PCP-70/30 (**a**) and expanded region from *m*/*z* = 750 to 1050 (**b**).

**Scheme 3:**
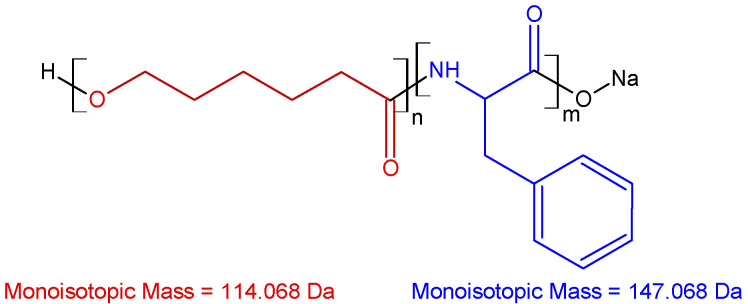
Hypothetical structure used for the MALDI MS characterization of PCP copolymers.

The obtained mass spectrum presented in Figure 4a, using the DCTB matrix for the sample preparation, revealed a multitude of peaks with a broad distribution and it was cumbersome to identify the associated PCP structures. The highlighted region between *m/z* 750 and 1050 values allowed a better observation of the MS peaks. The *m/z* values were generally associated with PCP structures having a variable number of CL and Phe units (Figure 4b). Thus, a main series of peaks was identified assuming the hypothetical structure presented in Scheme 3. The repeat unit was employed to report the CL/Phe monomer sequence in the PCP chains, neglecting that comonomer sequences may be randomly formed during synthesis and the end chains may be provided by both comonomers.

The main series, corresponding to the MS peaks with higher intensities were assigned using the following equation: *m/z = n* × *114 (CL)+ m* × *147 (PA) + 1 (H) + 16 (O) + 23 (Na-salt) + 23 (Na-adduct)*.

The attachment of the Na cations to the carboxylic end chains is common for MALDI ionization of PEA as it was also observed by others, e.g., in the work of Rizzarelli et al. [54]. Full assignment of the observed MS peaks belonging to this series may be performed using the theoretical *m/z* values from Table 3. For example, the *m*/*z* = 927 corresponds to a chain having 5 CL and 2 Phe units.

MS/MS fragmentation of the PCP chains was carried out to validate the direct structural assignment from the MS data and to identify potential CL-Phe sequences. The fragmentation analysis was performed using MALDI MS/MS with laser-induced dissociation (LID) that involves using a laser to dissociate precursor ions, generating fragment ions for structural analysis. LID fragmentation in LIFT mode is commonly used to study complex molecules, including proteins [55] and polyesters [56], offering detailed insights into their molecular structure.

**Figure 5:**
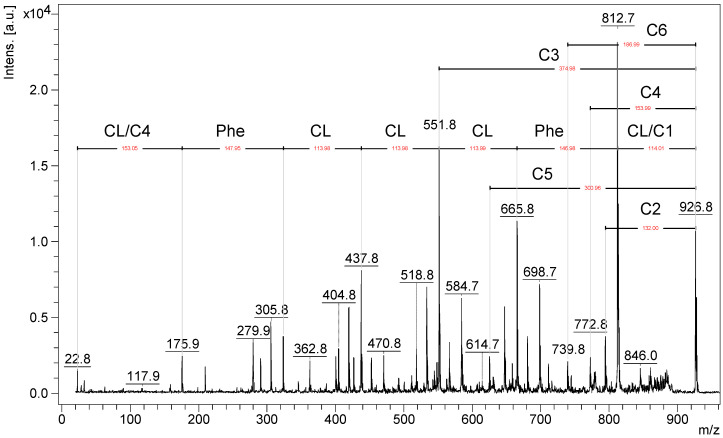
MALDI MS/MS spectrum of the [PA_2_CL_5_ + Na]^+^ parent ion and highlighted neutral losses (CL and Phe annotations stand for the moieties associated with the cleavage of the comonomer units; hypothetical structures of the neutral losses annotated C1–6 are given in Scheme 4 and Scheme 5).

The parent ion chosen for the fragmentation studies was associated with a linear chain containing 2 Phe and 5 CL comonomer units, annotated as [CL_5_Phe_2_ + Na]^+^, and having the *m*/*z* = 927. The obtained MS/MS fragmentation spectrum is presented in Figure 5.

**Scheme 4:**
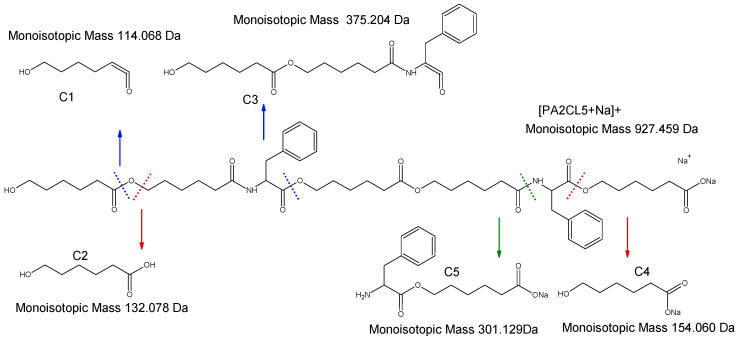
The hypothetical structure and theoretical monoisotopic masses of the neutral losses associated with the fragmentation processes of the [CL_5_PA_2_ + Na]^+^ parent ion obtained through MALDI MS/MS; the neutral fragments C1–C5 are associated with the MS/MS data from Figure 5.

The fragmentation of the linear chain proceeds through the cleavage of the ester and amide bonds, starting from both ends of the linear chain, and producing complementary fragments. The cleavage of the ester/amide bonds may proceed from the acyl or alkyl side through well-established mechanisms [54,56,57,58]. The main fragmentation pathway (Scheme 4) proceeds through neutral losses of comonomer units of 114 Da (fragment C1), corresponding to the loss of a neutral residue associated with the CL comonomer. This process corresponds to the cleavage of the ester bond from the acyl side, with fragmentation starting from the OH end chain. Another fragmentation pathway consists of an ester cleavage from the alkyl side, associated with the neutral loss of the 6-OH-hexanoic acid (Δ*m*/*z* = 132 Da, fragment C2 in Scheme 4).

Other fragments issued from the cleavage of the ester bond provided further insights into the structural assignment MS/MS spectrum. Thus, neutral losses containing phenylalanine residues were also observed. For example, the fragment ion observed at *m*/*z* = 551.8 resulted from a neutral loss of two CL and one PA comonomer units (Δ*m*/*z* = 375 Da, noted as C3 in Scheme 4). The fragmentation process proceeds through the cleavage of the ester bond on the acyl side.

**Scheme 5:**
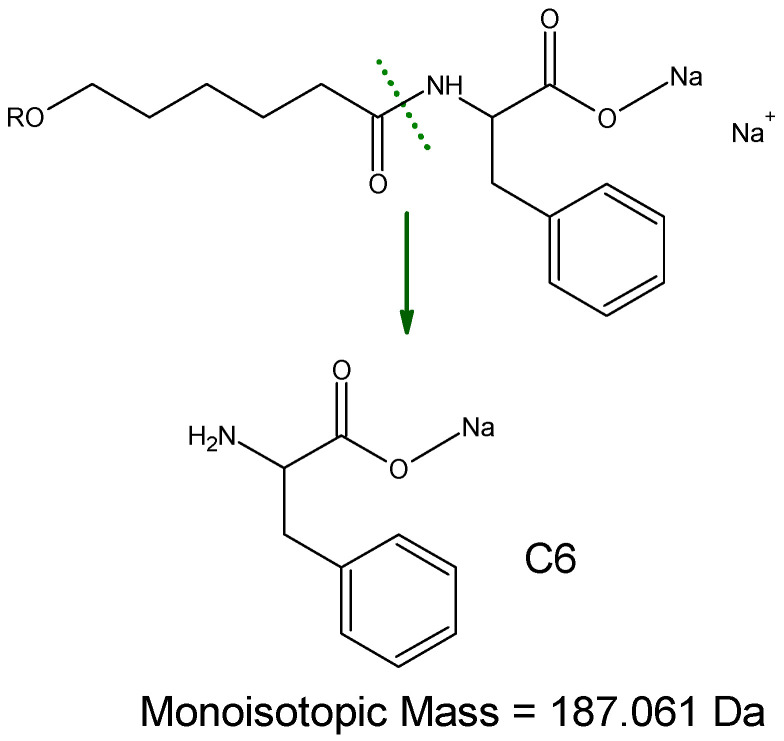
The cleavage of the terminal Phe moiety associated with the C6 neutral loss observed in the MS/MS spectrum of the [CL_5_PA_2_ + Na]^+^ parent ion.

Fragmentations occurred also from the Na-carboxylate end chain, being correlated with the neutral loss of the Na salt of the 6-OH-hexanoic acid, (Δ*m*/*z* = 154 Da, fragment C4). The loss of 154 Da also confirmed our hypothesis on the MALDI MS structural assignments that the PCP species terminated in a Na carboxylate salt. The fragmentation starting from this end chain further revealed neutral losses containing both CL and Phe comonomers. For example, the neutral loss of Δ*m*/*z* = 301 Da was associated with the structure containing the CL carboxylate salt and one PA unit (fragment C5). This process may be correlated with the cleavage of the amide bond, as depicted in Scheme 5.

The other peaks present in the MS/MS spectrum were issued through the above-mentioned mechanisms. The structure presented in Scheme 5 is only one of the possible structures that may be associated with the selected parent ion, as the sequence of the comonomer units may vary. Overall, the established fragmentation pattern highlighted in Figure 5 with a sequence of CL-Phe-CL-CL-Phe-CL-CL confirmed the intercalation of the Phe monomer units in CL sequences.

Among other possible sequences, the MS/MS fragmentation also pointed out the chains terminated in Phe. Thus, as highlighted in the MS/MS spectrum from Figure 5 the formation of the fragment ions at *m*/*z* = 739.8 was correlated with the cleavage of an amide bond and a neutral loss of 187 Da (structure C6), as depicted in the Scheme 5.

The MS/MS spectrum contained most probably more fragments issued from an amide cleavage. However, this fragment signaled the detachment of the terminal Phe unit connected to the PCP chain through the amide. This fact can be ascertained by the presence of the sodium salt of the terminal carboxylate function. Thus, the MS/MS analysis confirmed the contribution of the aminolysis reaction (Scheme 2 D2) to the formation of the PCP chains.

### 3.4. Thermal Properties

**Figure 6:**
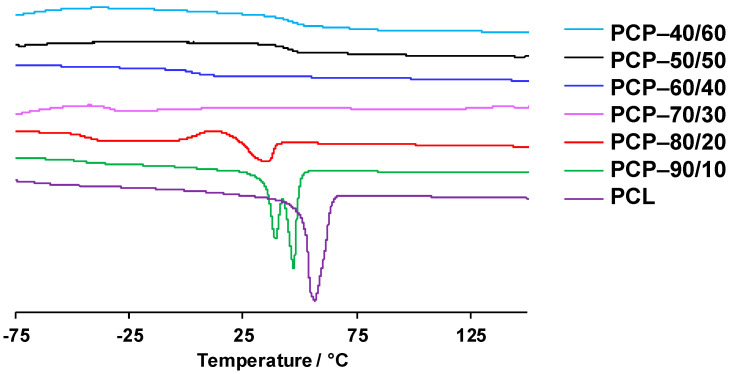
DSC thermograms obtained for the PCL and PCP-x/y copolymers.

The DSC results of PCP-x/y are given in Table 1 and the DSC curves are depicted in Figure 6. According to the DSC analysis, the synthesized PCPs were amorphous, except for the ratios PEA-90/10, and PEA-80/20, which displayed melting temperatures of about 47 °C and 35 °C, respectively. The semi-crystalline behavior of these PEAs was related to the increased content of CL. The amorphous nature of most PEAs may be correlated with the presence of mixed sequences. The disruption of PCL chains, caused by the incorporation of Phe units, increases the glass transition temperature (*T_g_*). Moreover, the formation of intermolecular hydrogen bonds between Phe units may contribute to the *T_g_* increase.

All PCPs exhibited a glass transition temperature (*T_g_*) above that of PCL (Table 1). As expected for the insertion of rigid units in flexible macromolecular chains, the *T_g_* increased when the Phe unit content increased. Typically, an increase in molecular weight is expected to correlate with a higher *T_g_*. However, when comparing PCP-60/40 to PCP-40/60, the opposite trend was observed: as the amide content increased, the molecular weight decreased significantly, with the *M_w_* dropping from 10,800 g/mol for PCP-60/40 to 3400 g/mol for PCP-40/60. This reduction in molar mass may have resulted from the deactivation of tetrabutoxytitanium, which depends on the increase of Phe content, as mentioned earlier. Despite this decrease in molar mass, the higher phenylalanine (Phe) content in PCP-40/60 resulted in increased *T_g_* values, suggesting that the additional hydrogen bonding from the amide units overrides the influence of molecular weight on *T_g_.* The melting behavior of the PCP-90/10 sample exhibited two distinct stages, which can be attributed to its composition of 90% caprolactone (CL) and 10% phenylalanine (Phe) by molar fraction. Due to the high content of CL in the copolymer, the initial melting stage was likely associated with the PCL sequences. The second melting stage was attributed to the PCL/Phe intercalated sequences.

Other PEA copolymers based on CL and β-alanine, glycine, and L-alanine amino acids [25,42,43,44,45] were characterized by lower *T_g_* values as compared to their counterparts of poly(ε-caprolactone-co-L-Phenylalanine), for similar comonomer ratios. This behavior may be correlated to the presence of the aromatic pendant functions that hinder the movement of the polymer chains and hence could account for the higher *T_g_* values noticed for the PCP studied herein.

**Figure 7:**
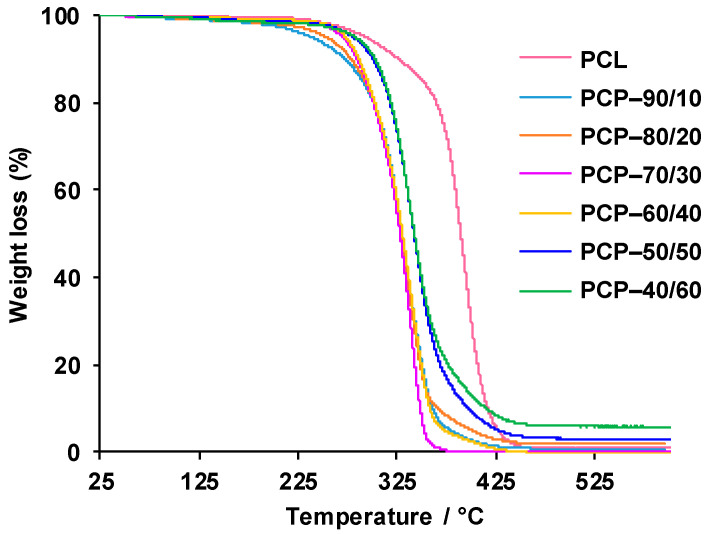
TGA curves of PCL and PCP-x/y copolymers.

The TGA analysis of the PCP-x/y copolymer series is depicted in Figure 7 and the characteristic 10% mass loss values are given in Table 1. The samples exhibited good thermal stability, as indicated by the 10% mass loss temperatures, which ranged between 272 °C and 303 °C. This behavior depends on the Phe content in the reaction feed of the PCP-x/y samples. Thus, the thermal stability of the samples was improved by the Phe presence due to the increasing influence of the associated hydrogen bonds between the polymer chains. However, the thermal stability may be correlated with the structure and the chain length. TGA analysis showed that the PCL homopolymer had better stability when compared with the copolymers. This may be correlated with the significantly higher *M_w_* (Table 1) as compared to the PCP copolymers. Generally, polymer samples with higher *M_w_* tend to exhibit greater thermal stability, as higher molecular weights result in stronger intermolecular entanglements, which require more energy to break down. Moreover, the stability of the samples is improved by the Phe presence due to the increasing influence of the associated hydrogen bonds between the polymer chains. The PCP-50/50 and PCP-40/60 samples were the most resistant among the synthesized PEAs since the fraction of amide units was the highest compared to the other PCPs.

## 4. Conclusions

In this study, a new series of PEAs was synthesized through a straightforward melt polymerization process, using varying amounts of L-phenylalanine and ε-caprolactone. The NMR and FTIR results were consistent with their intended chemical structure. The MALDI MS analysis revealed the presence of a PCP species as sodium adducts of the sodium salts of the PCP species and confirmed the intercalation of the Phe between CL comonomer units of the PCP chains terminated with hydroxyl and carboxyl functions. The established MS/MS fragmentation profiles of the sodium adduct of the PCP species revealed that the main fragmentation mechanisms involved are the cleavage of the ester and amide bonds. The observed fragments and their associated neutral losses allowed the assessment of the carboxylate end chains as belonging to both CL and Phe comonomers, thus confirming the occurrence of the aminolysis processes during synthesis. PCPs with molar ratios of 90/10 and 80/20 possess a semi-crystalline structure and exhibit increasing glass transition temperatures but decreasing melting points with increasing Phe content. Despite their relatively low molar masses, these copolymers demonstrated good thermal stabilities, making them promising candidates for various applications.

## Figures and Tables

**Table 1:** Synthesized PCP polyesteramides and their corresponding properties: weight-average molar mass (*M_w_*), dispersity (*M_w_/M_n_*), glass transition temperature (*T_g_*), melting point (*T_m_*), crystallization temperature (*T_c_*), melting enthalpy (Δ*H_m_*), and 10% mass loss temperature (*T_d,10%_*).

Sample CL/Phe *	*M_n_* (g.mol^−1^)	*M_w_* (g.mol^−1^)	*M_w_/M_n_*	*T_g_* (°C)	*T_m_* (°C)	*T_c_* (°C)	Δ*H_m_* (J.g^−1^)	*T_d,10%_* (°C)
PCP-40/60	2320	3400	1.47	47	−	−	−	303
PCP-50/50	3290	6900	2.10	44	−	−	−	301
PCP-60/40	4640	10,800	2.33	3	−	−	−	287
PCP-70/30	5110	9300	1.82	−34	−	−	−	285
PCP-80/20	14,490	32,900	2.27	−44	35	12	15	278
PCP-90/10	13,720	31,200	2.27	−57	47	−	58	272
PCL	51,180	143,300	2.80	−64	57	−	68	326

* CL/Phe molar ratio in the reaction feed for a specific sample.

**Table 2:** PCP trifluoroacetate triads and corresponding atom numbering (C corresponds to CL and P to Phe monomer units).

Triads	Structures and Atom Numbering
CCC	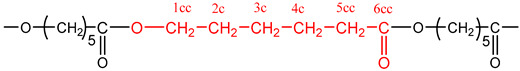
CCP	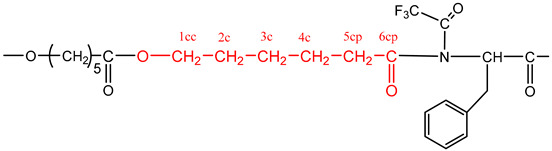
PCC	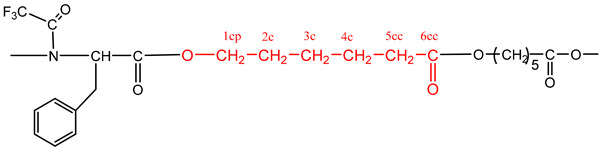
PCP	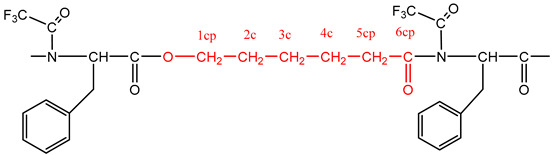
PPP	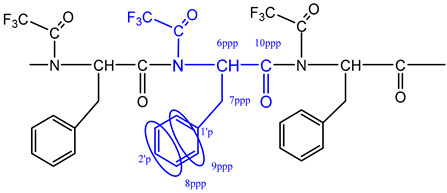
CPP	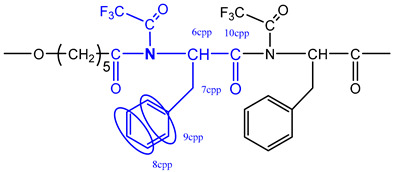
PPC/ CPC	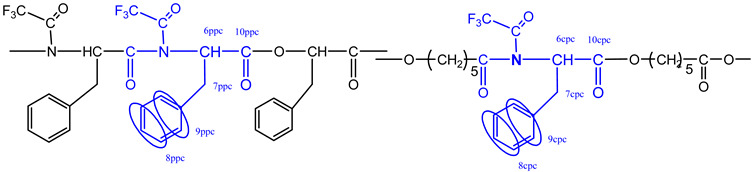

**Table 3:** MALDI MS *m/z* values employed for the structural assignment of the PCP chains.

	#PA *	1	2	3	4	5	6	7
#CL *		210 ***	357	504	651	798	945	1092
1	177 **	324	471	618	765	912	1059	1206
2	291	438	585	732	879	1026	1173	1320
3	405	552	699	846	993	1140	1287	1434
4	519	666	813	960	1107	1254	1401	1548
5	633	780	927	1074	1221	1368	1515	1662
6	747	894	1041	1188	1335	1482	1629	1776
7	861	1008	1155	1302	1449	1596	1743	1890

*—the number of CL and Phe units considering an increment of 114 Da and 146 Da, respectively; **—the starting structure of sodiated CL salt (*m/z = 114 (CL) + 1 (H) + 16 (O) + 23 (Na-salt) + 23 (Na-adduct)*); ***—the starting structure of sodiated Phe salt (*m/z = 147 (Phe) + 1 (H) + 16 (O) + 23 (Na-salt) + 23 (Na-adduct)*).

## Data Availability

The raw data supporting the conclusions of this article will be made available by the authors on request.
